# Secondary prevention of chronic health conditions in patients with multimorbidity: what can physiotherapists do?

**DOI:** 10.15256/joc.2016.6.82

**Published:** 2016-04-28

**Authors:** Sarah Dennis

**Affiliations:** ^1^Discipline of Physiotherapy, Faculty of Health Sciences, University of Sydney, Sydney, NSW, Australia

**Keywords:** Multimorbidity, multiple chronic conditions, physiotherapy

Multimorbidity is the co-occurrence of two or more diseases in an individual without a defining index disease [1, 2]. In developed countries, the prevalence of multimorbidity has been estimated from both general practice and population data [3, 4]. Data from general practices in Scotland found that 23% of patients had multimorbidity [3], whereas the prevalence of multimorbidity in Québec, Canada, was 46–51% in the general practice population and 10–13% in the general population aged over 24 years [4]. Australian data indicate that almost 40% of people aged over 44 years have multimorbidity, and this proportion increases to around 50% of those aged 65–74 years and to 70% of those aged 85 and over [5]. Data from a study of Australian general practice activity reported prevalence estimates for the most common combinations of chronic conditions [6]. Of the 12 most common combinations, the majority included conditions that can be positively impacted by physiotherapy interventions, such as low back pain [7], arthritis [8], chronic obstructive pulmonary disease [9], cardiac disease [10] and type 2 diabetes [11]. However, for some of these conditions, the uptake and access to physiotherapy interventions was suboptimal, especially in the primary care setting, due to poor referral from general practitioners (GPs) [12, 13] and/or restricted access to physiotherapy associated with workforce shortages, as well as high cost to the patient for private consultation.

In an invited presentation at the Australian Physiotherapy Association Conference 2015 in the Gold Coast, Queensland, I argued that clinical and academic physiotherapists, along with their academic colleagues in primary care, have the skills to lead research into effective interventions for patients with multimorbidity and to better define the role of physiotherapy, especially in the patient-centred medical home model of care [14]. In addition, I argued that there is a need to influence policy to provide improved and timely access to early effective physiotherapy interventions in primary care for people with, or at risk of developing, multimorbidity.

Physiotherapy researchers, similarly to their colleagues from other areas of healthcare, have focused their research on improving outcomes for patients with a specific single chronic disease and, as a result, there is limited published experimental research to determine the most effective approach for the management of multimorbidity. Physiotherapy research also tends to focus on interventions in secondary or tertiary care settings. A systematic review of interventions to improve the outcomes of patients with multimorbidity identified 18 randomized controlled studies, but half of these were interventions directed at patients with comorbidity, such as diabetes and depression, rather than multimorbidity [15]. There were six other studies in the review that reported on the effectiveness of patient-oriented interventions for multimorbidity (or comorbidity in one case), of which two were delivered by physiotherapists or occupational therapists. Reassuringly for physiotherapists, these interventions improved the functional outcomes for patients. 

A systematic review of chronic disease management in primary care using the chronic care model (CCM), as described by Wagner *et al*. [16], identified effective interventions for single chronic diseases [17]. Effective interventions include self-management support and multidisciplinary team care [17], i.e. interventions that non-GP healthcare professionals could provide effectively [18]; however, we need to know if similar approaches are effective for people with multimorbidity. While there is a need to undertake more multidisciplinary research to inform the most effective way for healthcare teams to manage multimorbidity in the patient-centred medical home, a key challenge is selecting or developing appropriate outcome measures [19–21]. 

A recent systematic review of the literature demonstrated an association between multimorbidity and functional decline [22]; functional decline was greater in patients with increasing numbers of chronic conditions and with greater disease severity. Furthermore, functional decline was greater for patients with cardiovascular and respiratory diseases than for those with other chronic diseases [22]. Physiotherapists are experts in providing interventions that reduce functional decline in older people [23, 24], and in people with cardiovascular [9], respiratory [9, 25], and neurological conditions [26]. A recent rapid review of the allied health literature indicated that if allied health professionals, such as physiotherapists, provided public health type interventions to prevent functional decline, there would be considerable savings to the healthcare system [27]. Interventions that addressed functional decline and that were singled out as having the potential to save money included falls prevention and reducing pain from osteoarthritis, which is a highly prevalent condition in patients with multimorbidity [6]. However, in Australia, there is some evidence for the inverse care law [28] in relation to access to allied health professionals such as physiotherapists. Data from the National Health Performance Authority show that the proportion of people who had access to an allied health professional (including a nurse) in the prior 12 months was 17% for those living in the outer metropolitan areas with lower socioeconomic status, compared to 26% for those living in the more affluent metropolitan areas, despite the higher prevalence of chronic disease in the outer metropolitan areas [29].

In 1999, Australia introduced the Enhanced Primary Care (EPC) plan [replaced by the Chronic Disease Management (CDM) plan in 2005] into the Medicare Benefits Schedule (MBS) to facilitate improved access to multidisciplinary team care. The Team Care Arrangements (TCA) provide five Medicare-funded allied health visits, but this will not be adequate to improve health outcomes for patients with some chronic conditions, and patients may not be able, or willing, to pay for additional treatment [30]. A review of Medicare claims for the CDM plan identified wide variation in the use of allied health services, and that the use of the plans by GPs was suboptimal [31]. Multimorbidity has a social gradient, with disproportionately more people from low socioeconomic backgrounds affected [2, 32]; consequently, these people are less likely to be able to pay for additional visits. Moreover, secondary prevention by physiotherapists for patients with multimorbidity in Australia is more likely to be hospital or community based, following a recent and expensive hospital admission [33]. These services are often oversubscribed and understaffed, have long waiting lists, and are difficult for some patients to attend [34].

If allied health professionals, including physiotherapists, are to be the “untapped potential” in the Australian healthcare system [33], then reversing the inverse care law and increasing access to effective physiotherapy interventions in primary care is an important first step to reduce the burden of multimorbidity and its associated functional decline. Physiotherapy researchers are challenged to take a lead and undertake more research into the effectiveness, and especially cost-effectiveness, of interventions to reduce functional decline and to improve health outcomes for people with multimorbidity. A collaborative effort, working with colleagues in primary care, is required to determine the most effective way to provide multidisciplinary interventions in a patient-centred medical home model of care.
